# Proteomics-based Model for Predicting the Risk of Brain Metastasis in Patients with Resected Lung Adenocarcinoma carrying the EGFR Mutation

**DOI:** 10.7150/ijms.92993

**Published:** 2024-02-25

**Authors:** Qiuhua Deng, Fengnan Wang, Lei Song, Liangyu Chen, Ying Huang, Zhihua Guo, Haihong Yang

**Affiliations:** 1 Department of Clinical Laboratory, the First Affiliated Hospital of Guangzhou Medical University, National Center for Respiratory Medicine, Guangzhou 510120, China.; 2 Department of Thoracic Oncology, the First Affiliated Hospital of Guangzhou Medical University, State Key Laboratory of Respiratory Diseases, National Clinical Research Center of Respiratory Disease, Guangzhou 510120, China.; 3 Tianjin Key Laboratory of Clinical Multi-Omics, Tianjin 300308, China.

**Keywords:** Lung adenocarcinoma, brain metastasis, proteome, EGFR mutation, risk prediction

## Abstract

**Introduction:** Epidermal growth factor receptor (EGFR) mutation is common in Chinese patients with lung adenocarcinoma (LUAD). Brain metastases (BMs) is high and associated with poor prognosis. Identification of EGFR-mutant patients at high risk of developing BMs is important to reduce or delay the incidence of BMs. Currently, there is no literature on the prediction and modeling of EGFR brain metastasis at the proteinomics level.

**Methods:** We conducted a retrospective study of BMs in postoperative recurrent LUAD with EGFR mutation in the First Affiliated Hospital of Guangzhou Medical University. Tissue proteomic analysis was applied in the primary tumors of resected LUAD in this study using liquid chromatography-mass spectrometry (LC-MS/MS). To identify potential markers for predicting LUAD BM, comparative analyses were performed on different groups to evaluate proteins associated with high risk of BMs.

**Results:** A combination of three potential marker proteins was found to discriminate well between distal metastasis (DM) and local recurrence (LR) of postoperative LUAD with EGFR mutation. Gene Ontology (GO) analysis of significantly altered proteins between BM and non-BM (NBM) indicated that lipid metabolism and cell cycle-related pathways were involved in BMs of LUAD. And the enriched pathways correlated with BMs were found to be quite different in the comparison groups of postoperative adjuvant therapy, tyrosine kinase inhibitor (TKI), and chemotherapy groups. Finally, we developed a random forest algorithm model with eight proteins (RRS1, CPT1A, DNM1, SRCAP, MLYCD, PCID2, IMPAD1 and FILIP1), which showed excellent predictive value (AUC: 0.9401) of BM in patients with LUAD harboring EGFR mutation.

**Conclusions:** A predictive model based on protein markers was developed to accurately predict postoperative BM in operable LUAD harboring EGFR mutation.

## Introduction

Non-small cell lung cancer (NSCLC) accounts for approximately 85% of lung cancers with a 5-year survival rate of less than 15%, and approximately half of NSCLC cases are lung adenocarcinoma (LUAD) 1-3. Brain metastases (BMs) are a common complication of NSCLC, especially in driver mutant LUAD. Epidermal growth factor receptor (EGFR) gene mutations are the most common driver mutations in Asian LUAD patients. There is a significant association between EGFR mutation and the risk of BMs at the time of initial diagnosis in advanced lung cancer, and there is a high adjusted odds ratio (3.83) between EGFR mutation and the risk of BMs at the time of follow-up after curative resection for LUAD 4,5.

BM often predicts poor prognosis in NSCLC patients with EGFR mutations 6. However, the molecular mechanisms and evolution of BM in lung cancer have not been extensively studied. Next-generation sequencing (NGS) has shown that NSCLC patients with BM have more copy number variations and alterations in genes encoding members of the cyclin-dependent kinase family, the SMAD family, and the superoxide dismutase 2 and PI3K signaling pathways compared with primary NSCLC patients 7,8. The driver mutations in EGFR, KRAS, TP53, and ALK were found to be highly concordant (>80%) between primary NSCLC and matched BMs 7, suggesting that there must be unique mechanisms underlying the high incidence of BMs in these driver-mutated lung cancers.

Due to the high incidence of EGFR BM, there is an urgent need to develop novel biomarkers to predict BM status and manage EGFR mutation LUAD. Current methods using genomic data to predict the prognosis of cancers such as LUAD BM are inadequate and inaccurate. Analysis of proteomic data can improve our understanding of cancer etiology and progression and may help us to find biomarkers for predicting BM in EGFR-mutant LUAD patients. In a study aimed at identifying circulating biomarkers based on proteomic data for early diagnosis and monitoring, some proteins, namely cathepsin F and fibulin-1, were found to be novel diagnostic biomarkers for BM in NSCLC 8. Other proteins have been found to promote BM. Other proteins have been found to promote BM by creating a metastatic environment: the Wnt-related protein CEMIP, which is enriched in brain-metastatic lung tissue and tumor-derived exosomes, and the blood-brain barrier transporters ABCB1, ABCG2, and MCT1, which are undetectable in microvessels of more than 80% of BM samples 9,10. In lung cancer cell lines, the reciprocal interaction between BASP1 and EGFR was found to facilitate EGFR signaling in brain-metastatic lung cancer 11.

Few proteomic biomarkers have been reported to predict BM in LUAD patients with EGFR mutation, and no therapeutic measures seem to reduce the risk of BM in these patients. Therefore, we focused on resected recurrent LUAD patients harboring the EGFR mutation and used data-independent acquisition (DIA) proteomics to analyze BM-related proteins in these patients.

## Material and Methods

### Clinical sample collection

To collect the records of patients with postoperative recurrence, we conducted a retrospective analysis. From January 2012 to November 2018, 1,360 consecutive patients with LUAD who underwent radical thoracic surgery at the First Affiliated Hospital of Guangzhou Medical University Hospital and were diagnosed as stage IA-IIIA LUAD with EGFR mutation by PCR or NGS were retrospectively identified. Patients with follow-up visits every 3 to 6 months were included. Baseline and clinicopathologic information was collected from those who met the inclusion criteria. Patients also underwent computed tomography (CT) of the chest and abdomen and magnetic resonance imaging (MRI) of the brain to assess tumor recurrence at each visit.

**Figure 1:**
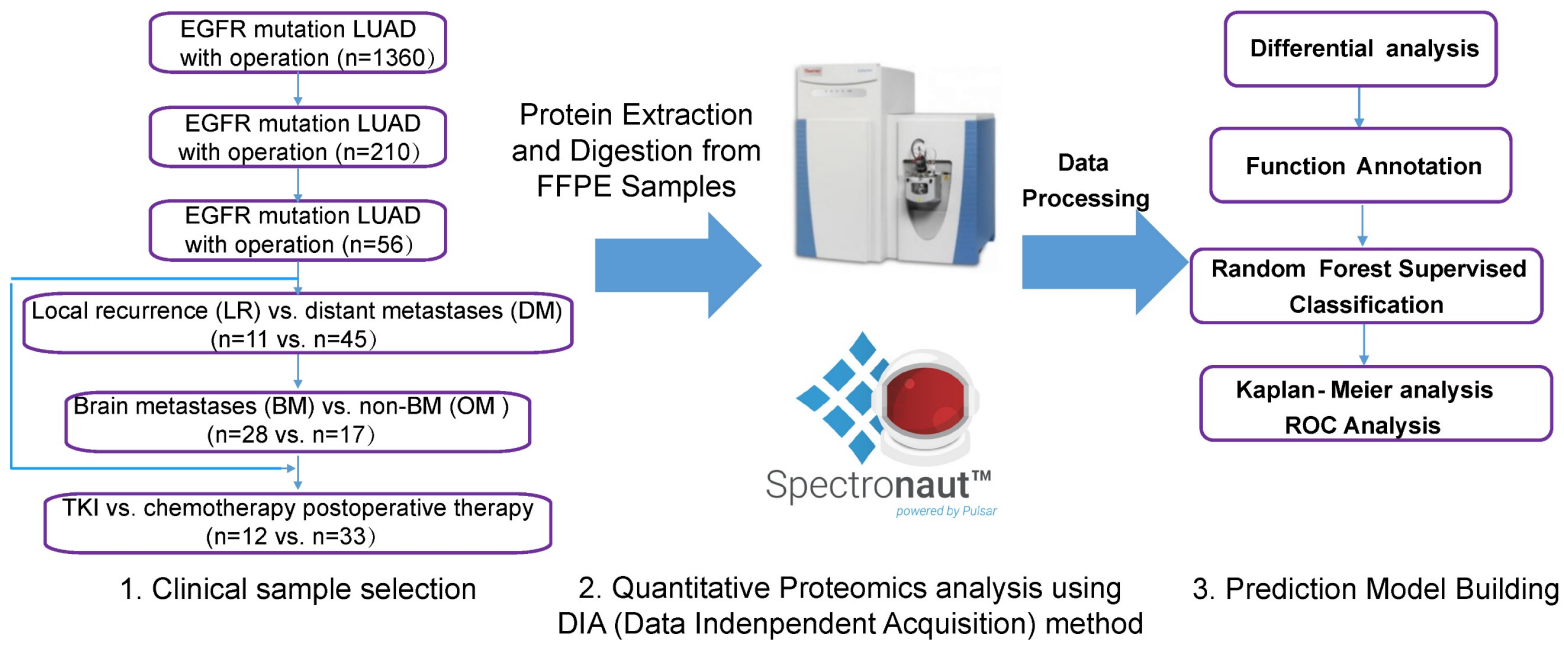
** Data collection of study participants and overview of the workflow in this study.** Clinical FFPE samples were selected from the LUAD with EGFR mutation cohort treated with different therapies (chemotherapy or TKI targeted therapy), which were divided into different groups (left); samples were analyzed by LC-MS/MS with DIA methods after protein extraction and digestion, and differential proteins were analyzed and a prediction model was built using random forest methods (right). LUAD: lung adenocarcinoma.

A total of 210 patients with recurrence after surgery were included in this study. Ultimately, 59 patients with detailed follow-up data and qualifying specimens (30 patients with BMs and 29 patients with non-BMs (NBMs) at first diagnosis of recurrence) were enrolled. Informed consent was obtained from all participants or their authorized relatives. This study was approved by the Ethics Committee of the First Affiliated Hospital of Guangzhou Medical University (No. CCTC2102). Patients were divided into different cohorts for further analysis, as shown in the left part of Figure 1, and the analysis process was shown in the right part of Figure 1.

### Protein extraction and digestion

Accurate assessment of tumor cellularity was determined by resecting the central portion of each tumor tissue block and subjecting it to hematoxylin and eosin (H&E) staining. Histologic evaluation of all tumor samples was performed independently by two board-certified pathologists.

Formalin-fixed paraffin-embedded (FFPE) samples (five serial sections, 5 µm thick) were first deparaffinized by two washes (10 minutes each) in 1 mL xylene at room temperature, followed by two washes in 1 mL absolute ethanol. Ethanol was completely removed and sections were air dried. Samples were resuspended in lysis buffer containing 4% sodium dodecyl sulfate and 100 mM Tris-HCl, pH 8.5, heated at 95 ^o^C for 60 minutes, and then sonicated with a 2 s on-3 s off cycle for 15 minutes. The SP3 protocol for protein digestion was performed manually as described previously 12. Briefly, 10 µg of extracted protein was added to tubes in a total volume of 10 µL lysis buffer.

### High-pH reverse phase liquid chromatography fractionation

To increase the depth of protein identification, peptide fractionation was performed using high pH reversed-phase liquid chromatography. Peptide mixtures were fractionated using a Waters XBridge BEH300 C18 column (250 × 4.6 mm, OD 5 mm) on a Shimadzu Prominence high-performance liquid chromatography (HPLC) system. 72 fractions were collected with a 90 min gradation (A: NH4OH in H2O, pH10; B: 80% ACN; 0-5 min, 5% B; 5-75 min, 5-80% B; 75-80 min, 80% B; 82-80 min, 80-5% B, 82-90 min, 5% B) and a flow rate of 1 mL/min and combined into 20 fractions. Each fraction was analyzed individually using the data-dependent acquisition (DDA) method as described below.

### LC-MS/MS

Equal amounts of iRT (purchased from Biognosys) were added to each DDA or DIA run on a Thermo Scientific U3000 nanoflow LC system, followed by a Q Exactive HF mass spectrometer. Peptide samples were eluted with loading buffer (2% ACN) and separated on a 150 µm ID × 30 cm column (C18, 1.9 µm, 120 Å, Dr. Maisch GmbH) using a 150 min gradient (A: 2% ACN, 0.1% FA; B: 80% ACN, 0.1% FA; 0-3 min, 3 to 9% B; 3-127 min, 9% to 63% B; 127-131 min, 63% B; 131-149 min, 63-3% B) at a flow rate of 600 nL/min. The spray voltage was set to 2,000 V in positive ion mode and the ion transfer tube temperature was set to 270.

For DDA, a 120K resolution MS scan at m/z 200 was performed, followed by triggering of the top 40 precursors. The MS AGC target was set at 3e6 or 40 ms of the maximum injection time generated by the Orbitrap mass analyzer (350-1,500 m/z). The MS/MS AGC target was set at 5e4 or 40 ms max injection time generated by higher energy C-trap dissociation (HCD) fragmentation at a resolution of 15,000 @ m/z 200. The normalized collision energy (NCE) was set to NCE 28% and the dynamic exclusion time was 16 s.

For DIA, a 60 K resolution MS scan was performed at m/z 200 MS and the AGC target value was set at 1e6 or 20 ms of the maximum injection time of the Orbitrap mass analyzer (350-1,500 m/z). The MS/MS AGC target value was set at 1e6 with an auto-set maximum injection time generated by HCD fragmentation at 30,000 resolution @ m/z 200. The NCE was set to NCE 28%. For proteome DIA MS runs, the fragment analysis was divided into 40 DIA isolation windows of different widths depending on the DDA search results. MS scans were also performed prior to each DIA cycle.

### Quantification of global DIA data

Hybrid spectral library generation was performed against the human UniProt database (July 2019, 20,365 sequences) using Spectronaut software (version 14.5.200813.47784) with default settings. DIA data were searched against the generated library using default settings. The acquired protein intensity matrix was normalized using the fraction of total (FOT) method. A fold change of 1.5 and a t-test p-value of 0.05 were set as cut-off values for differentially expressed proteins. Proteins with area under the curve (AUC) values greater than 0.8 were analyzed for associations with clinical outcomes. An unsupervised clustering heatmap was generated using custom R scripts and R packages. A predictive model was built using random forest after differentially expressed protein analysis and functional annotation. Figure 1 illustrates the workflow for this study.

### Survival analysis

In the absence of clinical data on overall survival, we chose disease-free survival (DFS) time, defined as the time from radical thoracic surgery to the time of recurrence of BM, as the endpoint. Survival curves were constructed using the Kaplan-Meier method, and the log-rank test was used to calculate differences between the curves. Hazard ratios (HRs) and their 95% confidence intervals (CIs) were estimated for each multivariate survival analysis using Cox proportional hazards models in the R survival package.

## Results

### Demographic and clinicopathological characteristics

A total of 59 patients were enrolled, 30 with BM and 29 without BM at first diagnosis of recurrence, with a median age of 59 years (ranging from 27 to 78 years). The postoperative pathologic stage was IIIA in 84.2% (50/59) of the patients.

We used the differential protein abundance data collected before metastasis to analyze which group of proteins might contribute most to metastasis. The number of identified proteins in the 59 samples ranged from 4,816 to 6,526. As mentioned above, we eliminated three samples with too few proteins (<5,800).

Of the remaining 56 tumor samples, all harbored common EGFR mutations, i.e., exon 19 deletion or p.L858R. Fifty of the 56 patients received adjuvant therapy: 33 (58.9%) patients received adjuvant chemotherapy alone, 12 (21.4%) patients received adjuvant TKI treatment alone, while 5 (8.9%) patients received both adjuvant chemotherapy and TKI treatment. In our samples, lung was the most common site of recurrence in addition to BM. The 56 patients were divided into two groups according to the site of recurrence (Figure 1): 11 patients with local lung recurrence (LR, lung metastasis only) and 45 patients with distant metastasis (DM, non-lung metastasis). The DM group was further divided into two groups: 28 BM (BM only and BM plus other types of metastases) and 17 other DM (ODM). Thus, the total patients were again divided into two groups based on the presence of BM: 28 BMs and 28 NBMs (LR+ODM). The demographic and clinicopathologic characteristics of the patients are shown in Tables 1 and S1. There was no significant difference in the development of BM between patients with different age, sex, EGFR mutation type or postoperative treatment (P > 0.05).

### Establishment of a model to distinguish local and distant metastasis

**Figure 2:**
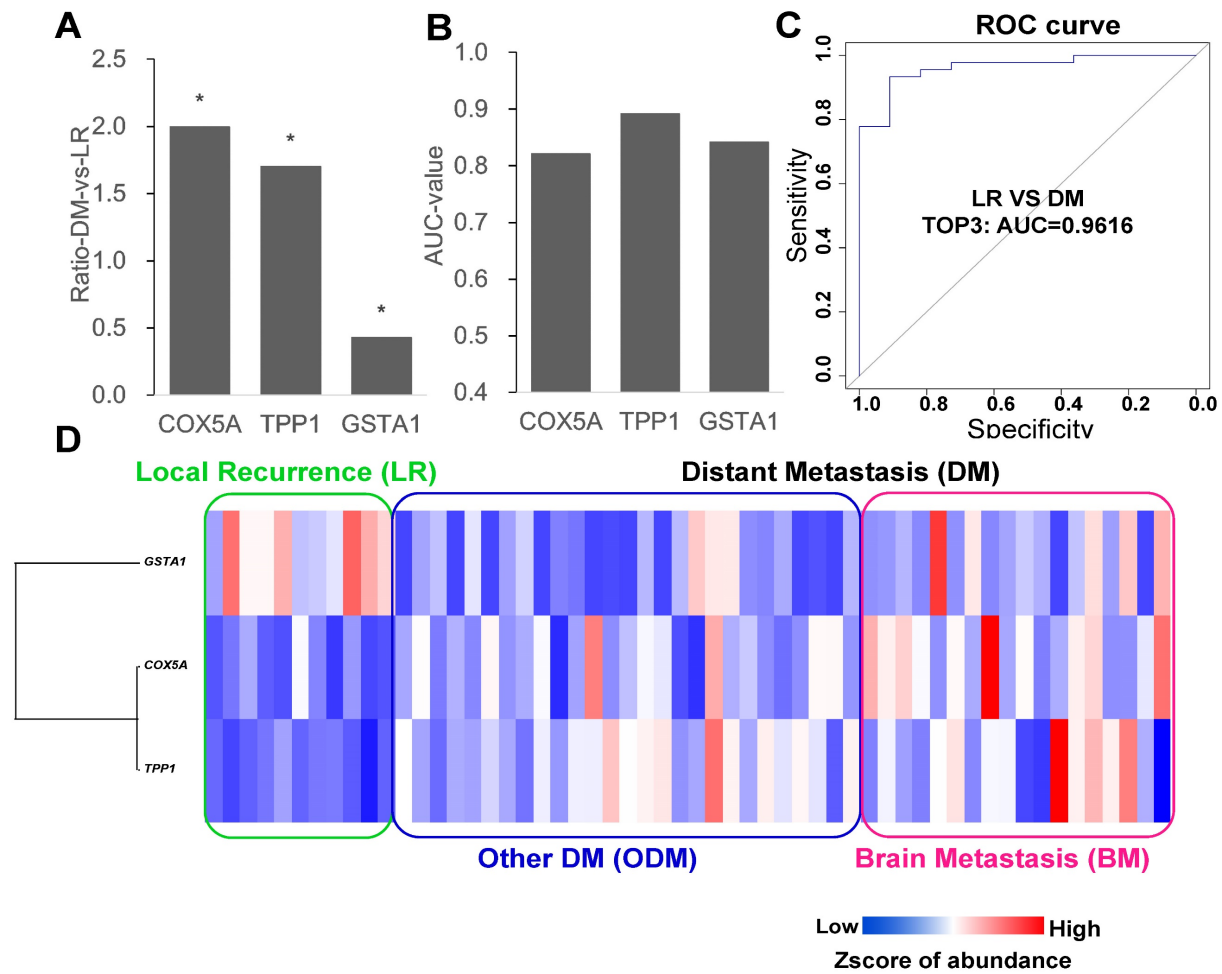
** Top3 proteins to compare local recurrence and distant metastasis. (A)** Fold change to discriminate LR from DM (other DM and BM) (Wilcox test: p<0.05, fold change >1.5). **(B)** ROC curve analysis to evaluate the discriminative power with AUC area of each significant change protein between DM and LR. **(C)** Combined top3 proteins got the AUC area at 0.9616. **(D)** Heatmap showed three proteins with different expression in LR and DM group. LR: local recurrence; DM: distant metastasis; BM: brain metastasis.

Each protein was considered to separate the LR and DM groups, and the proteins with the highest AUC values were selected as candidate markers. The top three proteins, TPP1, GSTA1, and COX5A, had AUC greater than 0.8, and only GSTA1 was downregulated (Figure 2A and B). Next, different combinations of proteins were tested to build ROC models, and the model with the top three proteins was the most stable model with an AUC value of 0.9616 (Figure 2C). An expression level heatmap of the top three proteins could well discriminate LR and DM groups (Figure 2D).

### Cell cycle-related proteins were associated with recurrence time

**Figure 3:**
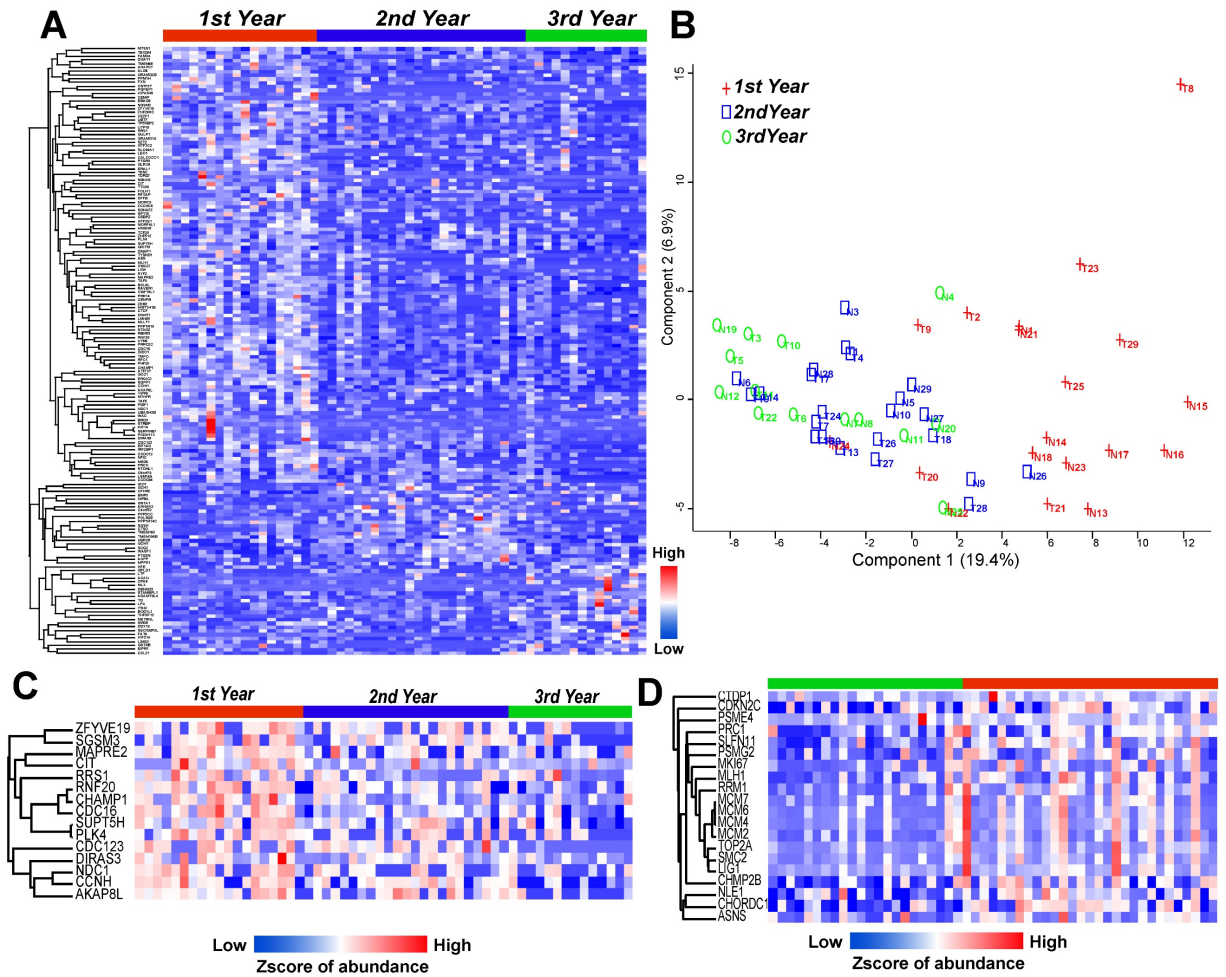
** LUAD prediction using cell cycle related proteins. (A)** Heatmap shows the significantly altered proteins among 1st to 3rd year recurrence samples (Annova test: p ≤ 0.05, fold change ≥ 2). **(B)** Principal component analysis (PCA) discriminated the 1st to 3rd year recurrence patients based on the differential proteins. **(C)** Heatmap showed the cell cycle related proteins with linear downregulation from 1st to 3rd year recurrence samples. **(D)** Cell cycle related protein with much higher expression in recurrent samples (red color) than non-recurrent samples (green color) with EGFR mutation. (Detected in the study of Xu et al).

To further evaluate the importance of recurrence-related proteins, the group was divided into three subgroups (1st year, 2nd year, and 3rd year or later) according to the time of recurrence, and indeed the highly expressed proteins were grouped into areas (Figure 3A). A PCA map showed that the 1st year group was mostly separated from the other two groups (2nd year and 3rd year groups) (Figure 3B). Although the 1st and 2nd year groups were partially mixed, the patients in the 2nd year group were closer to the 3rd year group. Thus, the patients were mainly ordered from left to right in the PCA map according to the year of recurrence. The 15 proteins annotated to the cell cycle pathway were highly expressed mainly in the 1st year recurrence group, and the expression decreased with time (Figure 3C). In addition, a study of LUAD samples with EGFR mutation by Xu et al13 showed that proteins in this pathway are highly regulated in the recurrence group (Figure 3D), indicating that the patients with a more highly regulated cell cycle pathway have a higher risk of recurrence.

### Random forest prediction model for LUAD BM

**Figure 4:**
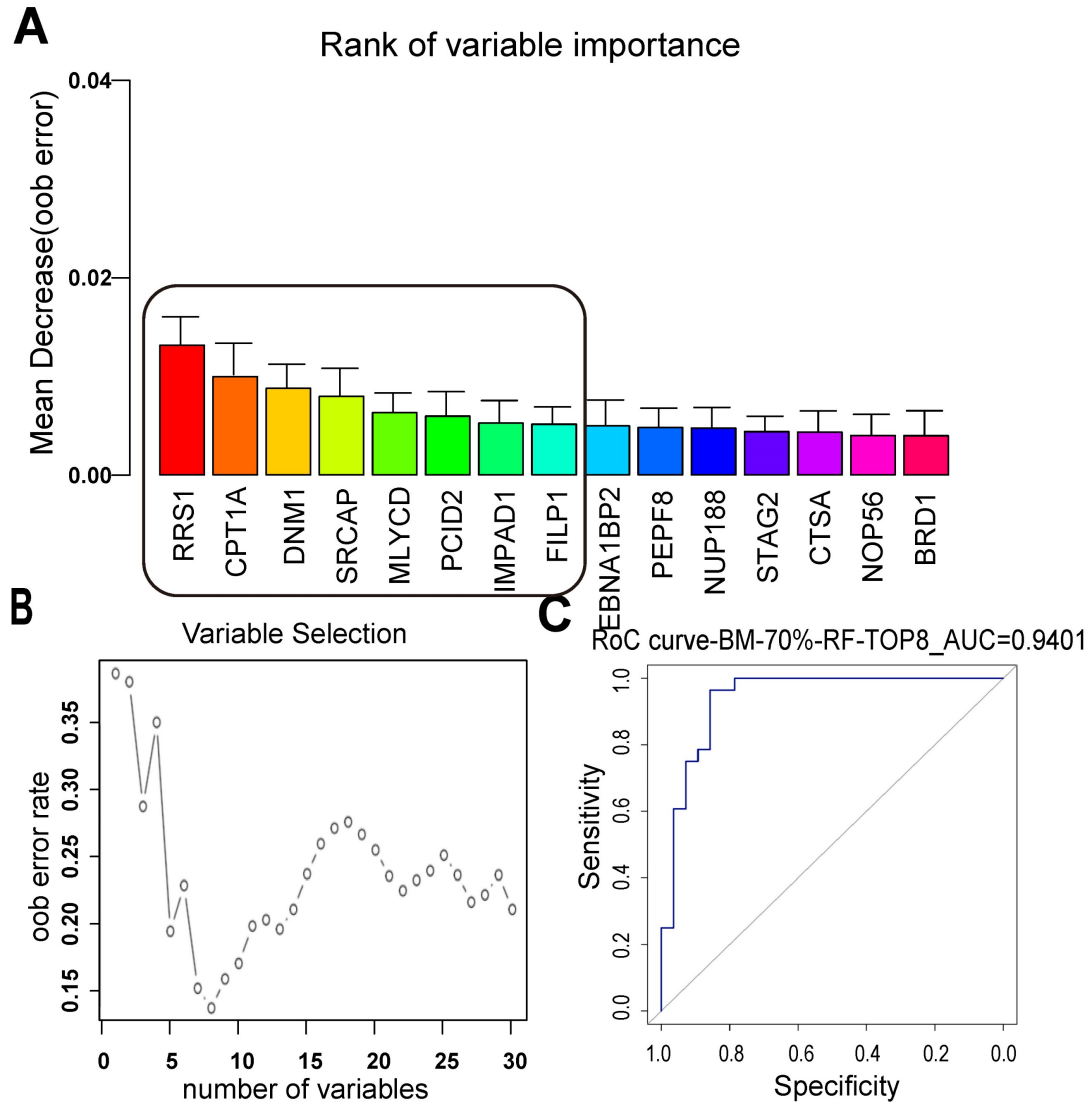
** LUAD BM prediction model built with random forest supervised classification. (A)** The 15 most important variables ranked by the mean decrease in accuracy estimated from 1,000 forests on out-of-bag (OOB) samples. **(B)** Selection of 8 variables in the predictive model based on the classification error rate on oob samples. **(C)** The constructed model for LUAD BM prediction with the top 8 proteins was with an ROC value of 0.9401. ODM: other distant metastasis; NBM: non-BM; oob error: out-of-bag error.

There was no significant relationship between the clinical factors and the occurrence of BM in the relapsed patients. Next, a random forest model was applied to separate the BM and NBM groups from another point of view. The top 15 proteins were listed, and the best 8 proteins were considered as RRS1, CPT1A, DNM1, SRCAP, MLYCD, PCID2, IMPAD1, and FILIP1 (Figure 4A). Among these proteins, CPT1A and MLYCD were included in the list of lipid metabolic pathways mentioned above. The curve of proteins used to construct the model showed that as the number of proteins increased, the error rate decreased and then increased again, with the lowest error rate corresponding to eight proteins (Figure 4B). The best model constructed with the top eight proteins had an ROC value of 0.9401, indicating that it performed well in separating the BM and NBM groups (Figure 4C).

### The lipid metabolic pathway was correlated with the development of BM

**Figure 5:**
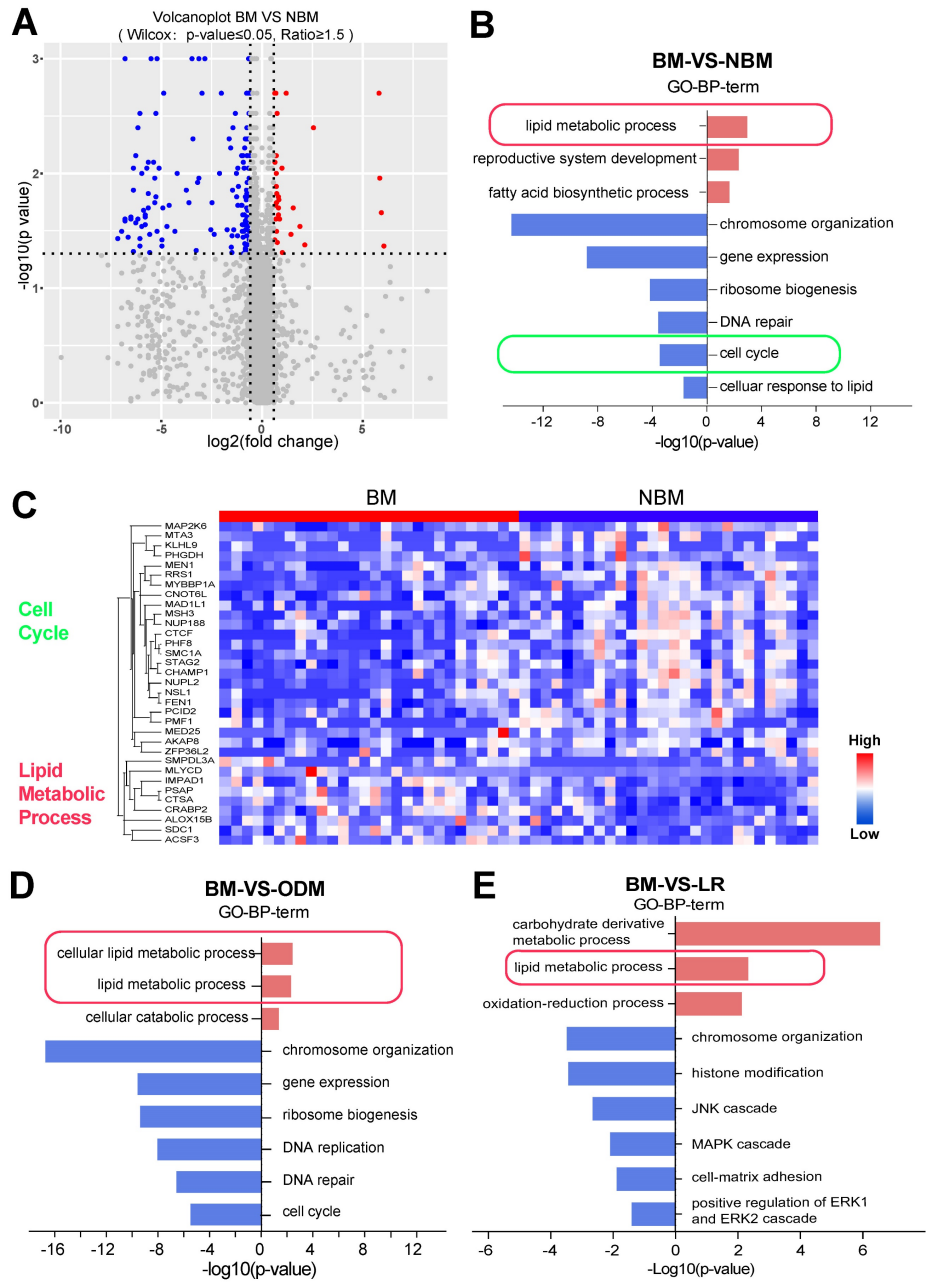
** Lipid pathways correlated with the development of BM. (A)** Volcanoplot showed that BM was well discriminated with OM (Wilcox test: p ≤ 0.05, fold change ≥ 1.5). **(B, D-E)** LUAD BM shows different changes in GO biological processing terms compared to NBM, ODM and LR based on significantly up- and down-regulated proteins. **(C)** Heatmap shows the differentially expressed proteins between BMs and NBM, with the cell cycle and lipid metabolism process related protein.

Further comparison of expression differences showed that the BM and NBM groups could also be well distinguished (Wilcoxon p-value ≤ 0.05, fold change ≥ 1.5) (Figure 5A). Gene Ontology (GO) enrichment was attempted based on the comparison between BM and the combination of NBM (Figure 5B). Proteins involved in lipid metabolism process were upregulated and those involved in cell cycle were downregulated in BM group (Figure 5B). The heat map shows the cell cycle and lipid metabolic process-related proteins between the BM and NBM groups (Figure 5C).

Since the NBM group contains LR and ODM groups, it was assumed that there should be differences between the LR and ODM groups based on the comparison with the BM group separately. Therefore, we evaluated the differentially expressed proteins from BM vs. LR and BM vs. ODM comparisons and found that there were many more down-regulated proteins than up-regulated proteins. LUAD BM shows different changes in GO biological process terms compared to LR or ODM group based on significantly changed proteins (Figure 5D and E). More down-regulated pathways were observed in both groups, but only the chromosome organization pathway was found in both groups.

Interestingly, proteins involved in the lipid metabolism process and related pathways were upregulated in both BM vs. LR and BM vs. ODM comparisons (Figure 5D and E). Notably, proteins involved in the fatty acid biosynthesis process were also upregulated in the ODM group, and those involved in the cellular response to lipid that consumes lipid were downregulated in the BM group (Figure 5D).

### Different postoperative adjuvant therapies were differently associated with incidences of BM

Here, the possible mechanisms of BM in the different postoperative adjuvant therapies, including TKI or chemotherapy treatment, were evaluated using proteomic data. The enriched GO pathways were quite different between the two treatment group comparisons (Figure S2A and B). In the patients who received chemotherapy, the downregulated proteins were significantly enriched in the chromosome organization pathway compared to the NBM group, and a number of proteins involved in nuclear-related pathways were also downregulated. The up-regulated proteins were enriched in lipid metabolism process and immune-related pathways. In the TKI group, upregulated proteins were enriched in pathways related to membrane changes, such as cell adhesion, membrane organization, and inflammatory response. In contrast, down-regulated proteins were enriched in pathways related to metabolites.

### Proteins associated with survival

All top 15 proteins were included in a proportional hazards model. Although all the proteins were within the differential proteins, not all of them had significant Cox p-values. The protein expression levels of MLYCD, IMPAD1, and FILIP1 predicted poor DFS in the patients with BM relapse (p < 0.05) (Figure S1A-C). Among the top eight proteins, only MLCYD, IMPAD1, and PRPF8 had both a significant Cox p-value and a significant survival p-value. In addition, CTSA only had a significant Cox p-value and FILIP1 and BRD1 only had a significant survival p-value. All eight proteins had ROC curve values greater than 0.67.

Among the top eight proteins, only one gene, MLYCD, was a differentially expressed protein (Table S2). Four proteins (CPT1A, DNM1, SRCAP, and IMPAD1) showed similar differential trends, and the remaining three are different. The reported data of 103 paired LUAD patients published by Xu et al. were used to validate the model in Table S3.13 The proteins DNM1 and IMPAD1 had a significant p-value for overall survival.

## Discussion

In previous studies, there were no related clinical characteristics that predicted the future occurrence of BM after resection of lung cancer in patients with EGFR mutations. Although we also found no significant difference in the development of BM among age, sex, or EGFR mutation type, we found that postoperative TKI treatment may lead to a lower incidence of BM in resected lung cancer patients. In recent years, research on the role of proteins in lung cancer progression has been gradually increasing 13-15. However, the application of proteomic data in the development of LUAD BM prediction models has not been reported. Some groups have developed proteomic or gene signatures of BM in lung cancer cell lines or body fluids such as serum 16-18. However, none of these studies developed proteomic models using human paraffin-embedded primary tumor tissues.

Here, we first found that a three-protein model (COX5A, TPP1, GSTA1) was more stable than models with other protein combinations, with an AUC of 0.9616, and could discriminate EGFR-mutant patients with local and distant metastases. The association of cell cycle with tumorigenesis and recurrence is well known 19. We found that 15 proteins in the cell cycle pathway were highly expressed mainly in the first-year recurrence group, with expression obviously decreasing with time. GO analysis of significantly downregulated proteins in different recurrent patients revealed enrichment in the cell cycle, which is somewhat different from the previous report by Xu et al. that cell cycle-related proteins have much higher expression in recurrent samples than in non-recurrent samples from patients with EGFR mutation.13 In our study, the cell cycle pathway was downregulated in BM patients. It was expected that the cell cycle would be inversely correlated with the incidence of BM in LUAD patients. The data from our sample set in this study first suggested that the BM patients could be distinguished from other relapsed patients.

Proteins with significant changes in abundance between BM and NBM samples were then subjected to random forest supervised classification to develop an eight-protein predictive model for BM in LUAD patients with the EGFR mutation (AUC: 0.9401). Researchers have continued to search for a good biomarker prediction model for lung cancer recurrence. The only gene reported to be predictive for BM recurrence was reported in resected NSCLC patients. A five-gene predictive model for BM recurrence using RNA sequencing data shows that high BM (AUC: 0.812) was associated with cell cycle and DNA repair pathways in operable LUAD 20. Our study is the first to develop a potential predictive model for LUAD BM patients with the EGFR mutation based on tissue proteomics data, which may fill the need for individualized precision therapy. To further demonstrate the significance of this new model for BM prediction, the association between these signatures and clinical outcomes was investigated using Kaplan-Meier analysis. Expression of three candidate proteins (MLYCD, IMPAD, and FILIP1) was correlated with BM-free survival, and two of them, MLYCD and IMPAD, were involved in lipid metabolism. Thus, these results were consistent with the identification of the lipid pathway as enriched.

Further analysis showed that more different proteins involved in lipid metabolism were associated with the occurrence of BM. The proteins up-regulated in the tumors in the BM group compared with the LR and ODM groups were enriched in the same GO term related to lipid metabolic process, indicating that up-regulation of lipid metabolic pathways, including fatty acid synthesis and oxidation (FAO), may be closely associated with BM recurrence. It has been found that the primary brain tumor glioblastoma multiforme relies on FAO for proliferation and that activation of FAO is achieved by high expression of acyl-CoA binding proteins or fatty acid oxidation enzymes 21,22. Recently, it has been reported that fatty acid synthesis is increased in HER2+ breast cancer tumors growing in the brain versus extracranial sites 23. However, to date, there are no similar reports for lung cancer. Our findings suggest that further investigation of the lipid pathway in BMs of EGFR-mutant lung cancer patients is warranted and that lipid metabolism may be the next potential preventive therapeutic target for LUAD BM.

We also found that BM tumors had downregulated immune pathways compared with LR tumors. Expression comparison of 770 immune-related proteins was performed between primary NSCLC and BM tissues, and the proportions of most immune cell subsets were lower in BMs 24, which is similar to the result in our study.

It has been reported that BM is more frequent in patients with tumors harboring EGFR mutations; NSCLC patients with tumors harboring exon 19 deletions showed a higher incidence of central nervous system involvement compared with those with tumors harboring the L858R mutation 25-27. Interestingly, we found that the prediction models of BM with different postoperative adjuvant therapies, TKI and chemotherapy, were associated with different types of differentially expressed proteins. In the chemotherapy group, down-regulated proteins were significantly enriched in the chromosome organization pathway and a number of nuclear-related pathways, and up-regulated proteins were significantly enriched in the lipid metabolism process and immune-related pathways. However, in the TKI group, up-regulated proteins were enriched in pathways related to membrane changes and down-regulated proteins were enriched in pathways related to metabolites. These results suggest that different postoperative adjuvant treatments may influence BM relapse in patients with operable LUAD carrying the EGFR mutation through different mechanisms. Thus, possible strategies to prevent BM occurrence may differ according to different postoperative adjuvant treatments in these patients.

In conclusion, a predictive model based on protein markers was developed and validated to accurately predict postoperative BM in operable EGFR-mutant LUAD patients. Furthermore, lipid metabolism was found to be strongly associated with BM recurrence in these patients. As the first article in the field of proteinomics prediction and modeling of EGFR brain metastasis, our study elucidates a potential correlation between brain metastasis and lipid metabolism abnormalities. Furthermore, we observe distinct mechanisms of brain metastasis associated with different postoperative adjuvant modalities.

## Supplementary Material

Supplementary figures and tables.

## Figures and Tables

**Table 1:** Demographic and clinicopathological characteristics (n=56)

	Characteristics	*N* (%)
Age (years)	≥60	27 (48.2)
	<60	29 (51.8)
Sex	Male	25 (44.6)
	Female	31 (55.4)
Stage	ⅠA	2 (3.6)
	ⅠB	2 (3.6)
	ⅡA	3 (5.3)
	ⅡB	2 (3.6)
	ⅢA	47 (83.9)
EGFR mutation	19 del	29 (51.8)
	L858R	26 (46.4)
	G719A	1 (1.8)
Smoking status	no	45 (80.4)
	yes	9 (16.1)
	unknown	2 (3.5)
Adjuvant treatment	no	4 (7.1)
	chemo	33 (58.9)
	TKI	12 (21.4)
	TKI+chemo	5 (8.9)
	NA	2 (3.7)
Brain metastases	total	28
	only brain	18 (64.3)
	brain and others	10 (35.7)
	Adjuvant treatment	27
	only chemo	18 (64.3)
	only TKI	5 (17.9)
Without brain metastases	total	28
	lung	13 (46.4)
	only lung	11
	lung and other	2
	others	15 (53.6)
