# Supplementary material for: Proteomics-based Model for Predicting the Risk of Brain Metastasis in Patients with Resected Lung Adenocarcinoma carrying the EGFR Mutation
Source: Int J Med Sci. 2024 Feb 25;21(4):765–74. doi: 10.7150/ijms.92993 (PMC10920840; doi:10.7150/ijms.92993)
Supplement: Supplementary file 1 — Supplementary figures and tables. [file ijmsv21p0765s1.pdf]

Supplement figure legends

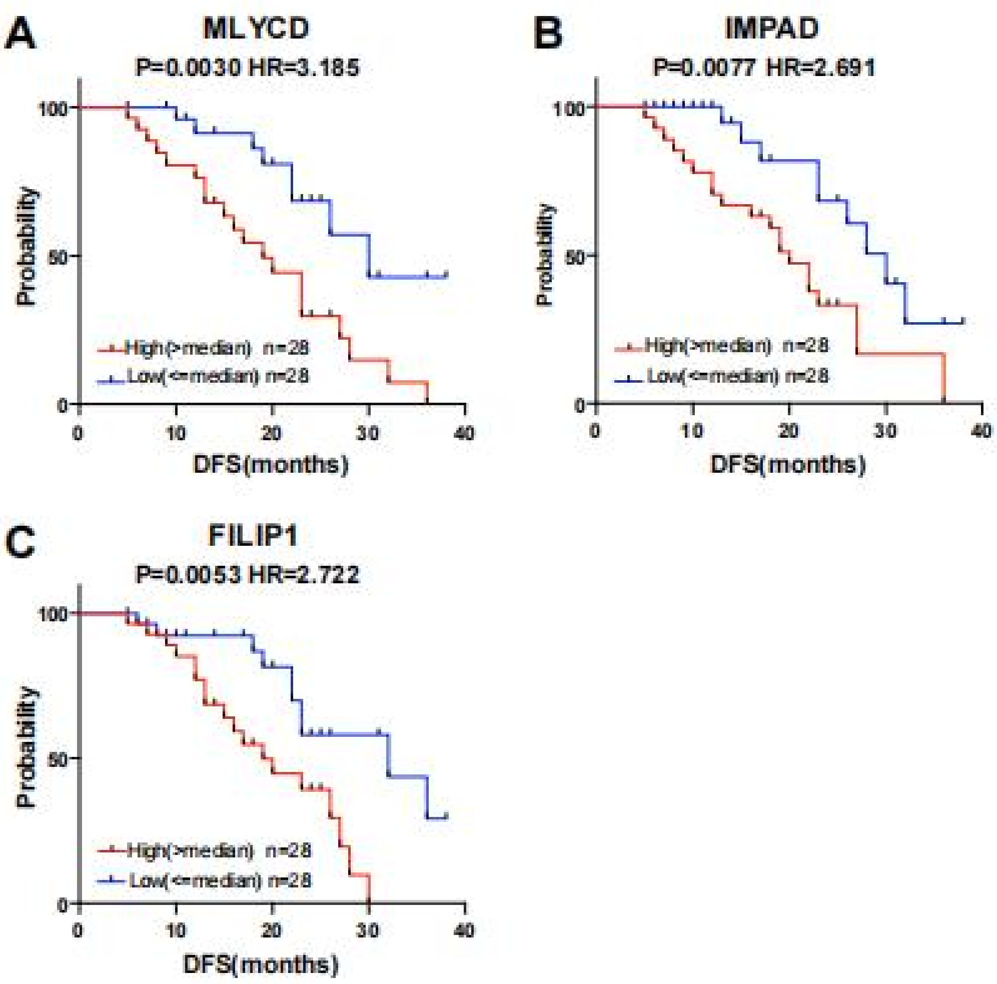

Figure S1. The survival of associated proteins. (A-C**)** The protein expression of MLYCD, IMPAD1 and FILIP1 predict poor DFS in the patients with BM recurrence (P<0.05). DFS: disease-free survival.

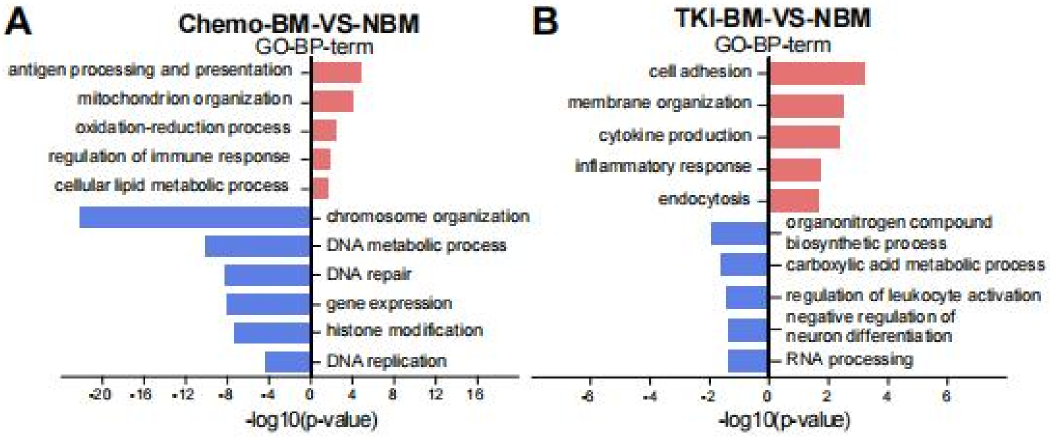

Figure S2. Differential Go changes between BM and NBM with chemotherapy and TKI target therapy. Significantly altered Go biological processing terms between BM and NBM with chemotherapy **(A)** and TKI **(B)** target therapy. Enriched upregulated (red) and downregulated (blue) Go biological processing terms based on significantly upregulated and downregulated proteins, respectively (wilcox test: $p < 0.05$, fold change > 1.5).

Table S1. The characteristics of patients with BM (n=28)

	Characteristics	*N* (%)
Age (years)	≥60	13 (46.4)
	＜60	15 (53.7)
Sex	Male	13 (46.4)
	Female	15 (53.7)
Stage	IA	1 (3.6)
	IB	1 (3.6)
	IIA	2 (7.1)
	IIB	1 (3.6)
	IIIA	23 (82.1)
EGFR mutation	19 del	16 (57.1)
	L858R	12(42.9)
Smoking status	no	22 (78.6)
	yes	6(21.4)
Adjuvant treatment	no	1 (3.6)
	chemo	18 (64.3)
	TKI	5 (17.9)
	TKI+chemo	4 (14.2)

Table S2. LUAD-BM prediction model gene list

Protein Groups	Q15050	P50416	Q05193	Q6ZRS2	O95822	Q5JVF3	Q9NX62	07Z7B0
Gene	RRS1	CPT1A	DNM1	SRCAP	MLYCD	PCID2	IMPAD1	FILIP1
Median-T	4.74	15.07	33.36	13.51	13.15	3.00	23.94	31.61
Median-N	20.46	22.74	20.64	7.67	5.73	4.84	12.06	8.57
Median-Ratio	0.232	0.663	1.616	1.761	2.296	0.620	1.986	3.690
Wilcox-pvalue	0.010	0.002	0.025	0.023	0.002	0.026	0.009	0.029
ROC	0.70	0.74	0.68	0.68	0.74	0.67	0.70	0.67
RF-TOP15-70%	1	2	3	4	5	6	7	8
Cox-pvalue	0.704	0.247	0.066	0.218	0.004	0.208	0.002	0.628
PH-pvalue	0.027	0.087	0.395	0.229	0.461	0.109	0.613	0.846
Hazard_Ratio	0.996	0.973	1.021	1.043	1.012	0.933	1.054	1.001
Surv-pvalue	0.941	0.454	0.131	0.260	0.003	0.174	0.008	0.005
Hazard_Ratio_surv	1.029	0.749	1.736	1.557	3.185	0.586	2.691	2.722

Table S3. CELL-LUAD-103-Paired-Wilcox (Detected in the study by Xu et al.)

Proteomics-T-vs-N	RRS1	CPT1A	DNM1	SRCAP	MLYCD	PCID2	IMPAD1	FILIP1
log2_FC_median	1.85	-1.13	1.37	2.10	-0.16	4.67	1.25	-2.53
FC_median	3.61	0.46	2.58	4.28	0.89	25.55	2.38	0.17
W_Pvalue	0.00	0.00	0.00	0.00	0.24	0.00	0.00	0.00
W_fdr	0.00	0.00	0.00	0.00	0.27	0.00	0.00	0.00
median_class-N	2.56	48.12	1.04	0.07	11.60	0.18	8.01	0.34
median_class-T	9.23	21.96	2.69	0.31	10.35	4.58	19.03	0.06
Cox_Pvalue	0.34	0.77	0.00	0.66	0.06	0.37	0.70	0.12
PH_Pvalue	0.94	0.20	0.75	0.20	0.78	0.86	0.62	0.85
Hazard_Ratio	1.02	1.00	1.16	1.12	0.95	1.03	1.00	0.34
OS-Surv_Pvalue	0.21	0.65	0.04	0.94	0.46	0.01	0.04	0.36
Hazard_Ratio_OS-Sur	1.66	0.84	2.30	2.97	0.75	3.21	2.32	0.70
DFS-surv_Pvalue	0.502	0.164	0.262	0.483	0.080	0.049	0.437	0.480
Hazard_Ratio_DFS-surv	1.19	0.70	1.34	0.83	0.64	1.67	1.23	1.20
RF-TOP8	1	2	3	4	5	6	7	8
